# Shifting dynamics of Sino-American competition in multi-aligned Middle East

**DOI:** 10.1016/j.heliyon.2024.e41053

**Published:** 2024-12-10

**Authors:** Arfan Mahmood, Muhammad Usman Askari

**Affiliations:** a Centre for South Asian Studies, University of the Punjab, Quaid-e-Azam Campus, Lahore, 54590, Pakistan; b Department of Political Science and International Relations, University of Management and Technology, Lahore, 54770, Pakistan

**Keywords:** Geopolitics, Competition, Regional stability, Engagement, Dominance, Middle East, US, China etc

## Abstract

The Middle East (ME) is one of the most volatile regions of the world where things can change in the blink of an eye. China, once an ordinary energy customer showing hardly any interest in the politics of the ME, has turned itself into a crucial player in rapidly transforming geopolitical landscape of the ME. The increasing footprints of China in the ME pose challenges to the US interests in the region. This article is an attempt to explore the dynamics of evolving competition between the US and China in the ME. It seeks to find out whether this alarmist narrative that the increasing footprints of China in the ME are a threat to the US interests and regional stability is well-put or obscures the deeper truth that China's increasing influence reflects a growing trend to multi-alignment in the ME. It further looks into the challenges faced by the US to better protect its interests in the face of rising influence of China in the region. This is a qualitative study which utilizes descriptive and analytical approaches to illustrate the dynamics of Sino-American competition in the ME. It is much easier to understand why Middle Eastern states are more attracted towards China as collaborating with a country other than the US helps them to break the shackles of US dependency. The suitable way for the US to solidify its position is through engagement than dominance.

## 1 Introduction

The ME has mostly been remained under the strong influence of the US until China has started presenting itself as a suitable contender for influence if not an alternative to the US in the ME. China has secured multiple victories one after another not just by enhancing its economic clout but also by convening high-level summits, negotiating peace agreements, and having military drills with perhaps the most significant ally of the US in the ME. Amidst the ongoing confrontation between Israel and Hamas, the only country which can count the preceding years a success is China [1].

The reproachment between two arch-rivals presided over by Xi Jinping in 2023 has been treated as a catalyst of world order transformation and decline of the US role in the ME [2]. China, once an ordinary energy client in the ME, has turned itself into a major contender for influence in the fast-changing geopolitical theatre of the ME in just a span of twenty years [3]. Just after a decade of the launch of Belt and Road Initiative (BRI), China's ties with the Middle Eastern countries especially with the Kingdom of Saudi Arabia (KSA) and the United Arab Emirates (UAE) have developed far beyond energy ties to a variety of other initiatives including infrastructure [4].

The Chinese blossoming ties with the KSA and UAE in particular reflect on the changing geopolitical dynamics and has implications far beyond the ME. These two countries have been emerging as crucial “middle powers” desiring to play their part in the transformation of world order and geopolitical contest between the US and China. The change in Chinese perspective on the ME is a symbol of its growing assertiveness and ambitious designs on the world stage [5].

These trends have been accelerated by the recent Russo-Ukraine and Israel-Gaza crises. Chinese reconciliation among the 14 Palestinian factions which ended 17 years long internal conflict among them is reflective of further world order transformation and decline of US role in the ME. They have strengthened the views that the US-led world order is in decline and the multipolarity in the ME is on the rise [6]. Nonetheless, the contradictions in Chinese position are also brought to focus by these wars. In spite of its growing presence and clout, China has maintained a very cautious or reserved approach to these conflicts. However, it is argued that the Sino-Middle Eastern ties remain somewhat shallow, driven by transactional communications and dilemma of lack of trust and understanding [7].

The increasing footprints of China in the ME in the shape of its active presence, to good numbers of analysts and policy-makers alike in the US, is a direct threat not only to the interests of the US but also to regional stability. Back in 2022, the then Undersecretary of Defense for Policy, Colin Kahl, stated Chinese intent and role in the ME in the following words: “The PRC pursues ties based solely on its narrow, transactional, commercial, and geopolitical interests … bypassing meaningful investments in regional security” [8]. This alarmist narrative is further seconded by the remarks of the General of US Air Force asserting that increasing clout of China in the economic, military, and diplomatic spheres in the ME is meant to “displace” the US from the region [9].

This US narrative is not well-grounded and is deemed misleading. China's increasing influence and presence in the ME is understood not to erode or displace the US influence, rather it reflects upon the growing trend of multi-alignment in the ME [10]. States in the ME are now more inclined to diversify their choices of partners as it helps them preserving their autonomy and self-interests in the best possible manner [11]. This shift to multi-alignment has made the way for Beijing to increase its footprints in the ME. In a similar fashion, it has provided the Middle Eastern states to develop stronger ties with the countries including India, Turkey, Japan etc.

The US tactics aimed at containing Chinese rising influence in the ME seem less likely to be successful especially when the Middle Eastern states resolutely look for new partners and welcome Chinese presence with open arms [12]. The US needs to revise its approach keeping in view the changing dynamics of the region [13]. So, it is imperative for the US to make best use of this multi-alignment trend in the ME by acting either as “broker” or “bridge,” and by pulling new stakeholders/countries in the region's security and economic well-being. The first option that can be utilized by the US is to “diversify” its own presence in the region by establishing a robust economic agenda which encourages more and more involvement of the private sector. The second option could be to invest in integrating its allies from both Europe and Asia well into the ME by enabling novel groupings. This tactic, on the one hand, can help amplifying the priorities of the US where the interests match/align, while on the other hand, it provides the ME with requisite resilience to dilute ME dependence on Chinese by presenting engagement alternatives [14].

This article is an attempt to reveal how the world order in general and the ME in particular is transforming under the growing geopolitical fragmentation and competition. The Chinese relations with the Middle Eastern countries especially with the Kingdome of Saudi Arabia (KSA) and the United Arab Emirates (UAE) reveal a burgeoning connection that is initiating to touch on geopolitical matters but has still to completely develop beyond its “economic infancy.” Further, these ties are intended to be analysed under wider geopolitical and geoeconomic advancements, especially structural changes in the global energy market, growing multipolarity, and increasing competition between the US and China. It seeks to unfold the mystery that whether China's rising influence in the ME is a direct threat to the US interest and regional stability or it reflects geopolitical pivot of the ME towards multipolarity/multi-alignment. So, the major query addressed through this study is: how does China's rising influence in the Middle East affect the US interests and regional stability of the Middle East and how does the US sustain and solidify its position in the fast-changing Middle East?

This is a qualitative study which utilizes descriptive and analytical approaches to illustrate the dynamics of Sino-American competition in the ME. Qualitative content analysis is the major aspect of research methodology of this study. Content analysis is adopted to explore the shifting dynamics of great power competition in the multi-aligned Middle East. This study primarily concentrates on the secondary sources of data collection. Primary sources in the form of interviews from the experts and ambassadors are emphasized. Secondary sources in the shape of government's policy documents, media reports, publications from security industry/institutions, and official statements from the executives of countries are mainly emphasized in the content analysis to answer the major queries of this study.

Qualitative content analysis is considered one of the most flexible methods of research with no strict directions of “when” or “how” to use it. It is adopted to analyse the changing dynamics of geopolitical competition between the US and China in the ME. The themes emphasized in this study for content analysis include ‘geopolitical competition’ between the US and China in the political, economic, social, and diplomatic realms, ‘multipolarity’ in the ME, and ‘changing dynamics’ of the ME etc. The contents emphasized for data collection of this study include ‘textual sources’ in the form of policy documents, media reports, institutional publications, and official remarks from the leadership of countries related to aforementioned themes have been focused. After codifying the data, the data has been analysed by employing both deductive and inductive methods, the application of which can be found in the data analysis section of the study.

This study is segregated into four parts. The first part informs about the key objectives and research method adopted for this study. The second part explores how the Chinese are increasing their footprints in the ME. The next section details about the US response to growing Chinese influence in the ME. The last part concludes the study with some policy recommendations.

### 1.1 Chinese growing footprints in the Middle East

The US has long been enjoying strong influence in the affairs of the ME whether its the region's economy, politics and security. In spite of its pivot away from the ME, the US still remains a major security provider of the region. Around 45,000 troops are still being maintained over more than a dozen installations in the region by the US which are supported by heavy armaments including fighter jets [15]. The US still remains the primary arms suppliers to the region which accounts for almost half of the entire region's arms imports. As far as the region's foreign direct investment (FDI) is concerned, the US is the major source despite a downward trend in trade volumes. The US has long been investing in diplomatic capital aimed at normalization of Arab-Israel conflict and to pacify regional tensions. Though the US still maintains an overwhelming influence in the region, today's ME is quite different from a decade old ME, with China being emerging as another major external player.

The decade of 2020 turned out to be very special and eventful for Sino-Middle Eastern relations. March 2023 witnessed China arbitrating between two arch-rivals Saudi Arabia and Iran, followed by an invite to Saudi Arabia and UAE for joining the BRICS [16]. There is growing debate at global level for purchasing of oil in renminbi [17]. All these developments raise concerns regarding the nature of Chinese blossoming ties with two of the most important Middle Eastern countries and question whether their ties are purely economic or involve geopolitical designs [18].

These events are an extension of already expanding Chinese ties with the KSA and UAE. Over the past two decades, China has registered itself as the largest importer of ME hydrocarbons [19]. The trade in energy remained the most crucial aspect of Sino-Middle Eastern ties. The region exports almost a quarter of its hydrocarbons to China only and it seems very likely that this trend will continue in the years to come. This cooperation got further expanded into infrastructure projects with the launch of Belt and Road Initiative (BRI) in 2013 through which the KSA and UAE collaborated with Chinese companies in variety of ports and industrial zones projects to strengthen their hold on world trade routes. However, this economic cooperation did not remain limited to energy trade and infrastructure rather its expanding to digital infrastructure, high-tech, and a diplomatic offensive aimed at promoting Chinese norms and narratives worldwide [20]. Consequently, Sino-Middle Eastern ties set of to diversify by emphasizing variety of sectors including emerging technologies, logistics and supply, and strategic minerals and renewable energies.

As far as the cooperation in renewable energy is concerned, Chinese enterprises are providing all kinds of support to the UAE to construct two massive solar energy projects [21] – Muhammad bin Rashid Al Maktoum Solar Park and Noor Abu Dhabi solar park. China also established “Silk Road Fund” which acquired almost half of the stakes in ACWA power, KSA's renewable-energy firm [22]. It is the KSA's major investment company in renewable-energy projects both within and beyond the region. In order to diversify its economy, KSA has collaborated with Chinese enterprises in many sectors, notably in electric cars sector. A joint venture of worth $ 5.6 billion was signed between Riyadh and “Human Horizon,” a Chinese electric vehicle making firm, which was aimed at establishing a research and manufacturing units in the Kingdom in 2023 [23]. Another venture was concluded between China's EV start-up Enovate and KSA's Sumou Holdings worth $ 500m in 2022 to develop a manufacturing unit in the kingdom [24]. The recent years have witnessed huge investment flows in the region's two most significant monetary funds – Mubadala of the UAE and Public Investment Fund of the KSA – to launch new franchises in China for targeting the Chinese market [21].

The AI and emerging technologies are one such area where the Sino-Middle Eastern cooperation could be problematic and potentially challenging for the West in general and the US in particular. As part of their drive to maximise investment in digital economy, both these gulf countries have accelerated their efforts to cooperate with Chinese firms and research organizations. In cooperation with Huawei and Alibaba, both these gulf countries developed some segment of their tech infrastructure ranging from 5G services to the development of large data centres. A new “cloud region” was launched in Riyadh by Huawei in September 2023 which was intended to support government services [25]. Further, the academic institutions such as “King Abdullah University of Science and Technology” in KSA and “Muhammad bin Zayed University of Artificial Intelligence” in the UAE invited top Chinese academics of AI research institutes to bolster their emerging technologies and AI potential. Besides leading ambitious initiatives to establish “Arabic large language models and AI applications,” MBZAI is comprised of 40 percent of its faculty by Chinese scholars. Moreover, G42, the best AI firm of the kingdom, collaborated extensively with Chinese enterprises to develop vaccine for COVID-19 epidemic and launching of the “Emirati Ministry of Health's genomics program” [26].

These exchanges have heightened the US reservations that Sino-Middle Eastern ties could provide easy access to China to the sensitive US technologies. A case worth highlighting here is the purchase of Nvidia chips by the kingdoms' research institutions engaged in AI research. The export of these chips produced by the US is prohibited to China. There is also anxiety among the US policy circles that the large-scale induction of Chinese high-tech in the region's digital setup could be utilized for intelligence gathering given the US extensive military ties with the ME states [27]. It is suitable to mention here that it is not only the Chinese firms that are partnered with these ME states across all these areas. Rather, its the US enterprises like Microsoft and Open AI that continue to be the primary choice by the ME states in their strategic projects. The kingdom's firms are bit reluctant to prefer Chinese companies over the US companies as they deem the US firms more innovative and reliable. But a lot of other advantages are retained by the Chinese firms. For instance, their price mechanisms are more competitive and they are more cooperative to technology transfer and localise production than the US counterparts. Even more striking is the connotations of the Sino-US tech war which has left very few business opportunities to Chinese firms overseas owing to the pressure the US places on its allies not to make use of Chinese technology. Resultantly, the ME states are provided with more bargaining chip for imposing heavier localisation clauses. As far as data-privacy standards are concerned, Chinese tech firms have less stringent data-privacy measures than the US firms.

China's growing economic clout in the region has attracted the US attention and is conceived as the biggest threat to the interests of the US in the region. The last decade has witnessed the Chinese trade volume with the region increasing by about 40 %, driven primarily by its growing demand of energy and the ME hunger for Chinese manufactured items [28]. The volume of Chinses investments in the region has increased manifolds as they are usually with no strings attached unlike the US [29].

Besides expansion of economic ties, Sino-Middle Eastern relations have witnessed expansion in political and security realms breaking away with China's conventional reserved strategy towards ME. The regions' two most important countries, KSA and UAE, have revised and upgraded their diplomatic staff towards China. For instance, the UAE appointed Khaldoon al-Mubarak, the president's advisor and CEO of Mubadala, as the kingdom's special envoy to China. In a similar fashion, the KSA appointed Khalid al-Falih, former CEO of Aramco, to lead kingdoms' relations with China. Recent decade has witnessed significant increase in high-level official visits to and from China. This upgradation of diplomatic ties reflects the growing significance of the relationship for all parties.

In the diplomatic realm, Chinese have surprised the international community by successfully mediating between Iran and Saudi Arabia. Even more surprising is its strategy to engage Midde Eastern states in multilateral arrangements/forums. Chinese, for example, have tried to attract some of the regional states including the ones in close partnership with the US into its own security and economic arrangements such as the BRICS and the SCO [30,31]. Though these groupings are quite different from the US formal alliances, but being part of them is an indicator that the regional states are interested to diversify their alignments. This is verified by the intent of the US partners such as KSA and UAE to seek close bilateral relations with China which is reflected through frequent high-level diplomatic visits [32].

The recent diplomatic move of China to broker a peace deal among 14 Palestinian factions which ended 17 years long internal conflict among them underscores Chinese increasing clout and presence in the ME. This deal is anticipated to contribute towards regional peace, security and stability but its impacts are still far to be seen. The Chinese veto to the US sponsored United Nations Security Council (UNSC) resolution in March 2024 over Gaza conflict is reflective of its growing engagement in the ME and the challenges it presents to the US in the region [33]. Moreover, it throws light on the changing approach of China towards the ME, the region's growing significance in Chinese foreign-policy agenda, and the changing dynamics of the region's countries' ties with each other and the US as well. This intensifying involvement of China in the ME demonstrates a greater shift in its geopolitical strategy towards the region.

Chinese response to Gaza issue helps clarifying its intensions further. Wang Yi, Chinese Foreign Minister, had a stop-over in Egypt and Tunis during his six-nation trip in January 2024 to clarify and promote Chinese position on the issue. Along with his Egyptian counterpart, he emphasized on the necessity of immediate ceasefire, and raised his voice and support for Palestinian national rights in Tunis [34]. The Chinese government was pro-active to send Zhai Jun, its special envoy for the ME [35], immediately after the start of Israel-Gaza crisis, and issued a policy document for the resolution of the conflict [36]. The Chinese took Israel-Palestine crisis as an avenue to further its involvement in the region. Moreover, China has long been presented itself as a mediator to resolve this crisis [37].

The limitations of Chinese influence in the region are also exposed through its response to Gaza crisis. It is argued that Chinese diplomatic attempts in this crisis are nothing but rhetorical. The Chinese proposed solution to the crisis is widely considered as not different from the previously proposed solutions of this crisis and hardly offers novel ideas that could help facilitating a settlement. Moreover, it seems that the Chinese leadership has not been actively involved in “concerted diplomacy” with the parties for diffusing the conflict.

The Chinese response to Gaza crisis is observed both with caution and geopolitical lens. It is argued that China might not have qualified as a ‘credible’ peace broker in the Israel-Palestine issue, at least by the Israeli authorities. While Chinese officials disagree and claim to be play as a neutral mediator in this crisis. China has been deemed bias and not appreciated by few for not condemning October Hamas attack on Israel and condemning the assassination of Ismael Haniyeah [38]. Beijing's role in the reconciliation among 14 Palestinian factions is seen with scepticism in the West and considered detrimental to the peace prospects between Israel and Palestine [39]. While China claims itself to be a force for peace.

The Chinese reconciliation among 14 Palestinian factions has earned significant weight and influence for Beijing in the ME. Both the US and China are tied in a geopolitical competition in the ME which has intensified because of growing Chinese influence in the region. The Chinese response to Israel-Gaza crisis underscores a major transformation of its foreign policy approach to the ME- “from ‘hedging’ by maintaining positive relations with regional actors to ‘wedging’ or attempting to exploit space between the US and its partners and allies in the ME” [40].

The security cooperation of China with the ME remained almost non-existent until the start of 21st century. But this security cooperation has started to transform. The People's Liberation Army (PLA) of China developed its very first miliary base in Djibouti back in 2017 [41]. The Chinese navy since then has been involved with the region in port calls and joint naval exercises. KSA holds joint military drills with Chinese PLA annually and hosts military officials from China [42]. The defence organizations of China have begun to make inroads in the region which is still overwhelmingly dominated by the West in general and the US in particular. Both the kingdoms, for instance, have imported military drones from China back in 2010. They also imported a specific technology (CH4 and Wing Loong) from China which the US is reluctant to provide owing to the concerns that it might undermine the “Missile Technology Control Regime” [43]. In the recent defence shows conducted by both the kingdoms in 2024, the pavilion hosted by China was much bigger than the US [44]. Both the kingdoms have sought Chinese cooperation to establish their own national defence sector, and since 2017 they have cooperated with China to jointly develop and manufacture combat drones and ballistic missiles in the region.

The US is quite sceptical of these developments. The US intelligence in 2021 showed concerns and indicated that Chinese may be establishing a covert military installation in Abu Dhabi's Khalifa Port [45]. Based on this intelligence report, the US suspended negotiations with the kingdom for the supply of F-35 fighter jets [46]. In 2023, there were rumours that Chinese were in discussion with Omani government for the possibility of military base in Oman [47]. The US responded by setting a potential Chinese base in KSA as a red line.

The Chinese mediation in the diplomatic reproachment between two arch-rivals, Iran and KSA, is considered highly unusual in the ME, a region which is conventionally dominated by the US. Against the backdrop of growing unease about the nuclear program of Iran, this diplomatic reproachment between two arch-enemies represented a crucial step towards regional de-escalation.

### 1.2 The changing Middle East

While looking at the greater picture, Chinese changing behaviour is only one part of the bigger trend which is unfolding in the ME. The Middle Eastern countries have embraced not just China but many other rising powers which has created a blend of overlapping partnerships with multiple extra-regional actors. Visualised from a greater perspective, Chinese active role in the region looks less like a “push” by China to diminish the US clout. Rather, it looks more like one part of the greater geopolitical shift which has largely been driven by the states of the region.

To diversify the options, the Middle Eastern states have not only built economic ties with China but also sought stronger economic linkages with India and retained their trade relations with the European Union (EU). India has emerged as another major trading partner of the ME recording 20 % increase in its trade only with Gulf Cooperation Council (GCC) countries in just a span of a decade [48]. Further, India has strengthened its relations with the UAE and Israel by participating in I2U2 minilateral. India has collaborated with them in diverse sectors including agriculture, infrastructure, and high-tech [49]. This reflects that China is not the only country targeted for investment in infrastructure by the ME countries. The purchase of Israel's Haifa Port by Adani Group in $1.2 billion is a glaring example of that [50,51].

In security realm, China is not again the only country sought after by the ME. South Korea is another preferred country after the US and China for the purchase of arms. A good number of regional states including KSA, UAE, and Egypt have negotiated arms sales pacts with South Korea. With these pacts worth billion of dollars, its market share is anticipated to grow significantly in the years to come [52,53]. Some other regional states have concluded defence cooperation pacts with South Korea recently which emphasize joint military drills. This has provided South Korea with reasonable security presence in the ME [54,55]. Last but not the least is the role of Russia as the regional arms supplier. Though there is a temporary disruption in arms supplies from Russia to the region because of its war in Ukraine, the region continues to involve Russia over energy and security matters of the region.

The entry of multiple external players in the ME reflects that perhaps its not meant to replace the US rather it shows the preferences made by the regional states to act in their own self/national-interest. This changed geopolitical setting where regional states are provided with multiple options is quite unique in the context of the ME. The Middle Eastern states seem much more interested to work with diverse group of partners rather than working only either with the US or China. This reality is supposed to be kept in consideration especially by the US while making foreign-policy decision for the ME.

### 1.3 The US response

The US response to the changing geopolitical realities of the ME, however, has not been considered up to the mark and fallen short of meeting its objectives. Its strategy is described as “triangulatory” having three key pieces. The first major element of its strategy is to “reassure its partners of its commitment and dedication to them.” In order to demonstrate its commitment to its partners, the US has further strengthened its military presence in the ME and carried on with arms supplies to them particularly after October 7. The second crucial piece of its strategy is to “continue with the objective of seeking expansion of Abraham Accords.” It is assumed that the addition of more and more countries in this particular network will help resolving Arab-Israel conflict [56]. Given the current state of affairs regarding Israel-Hamas conflict, this policy has not seemed working effectively.

The third and last piece of its strategy is to “pressurise countries to either choose the US or China.” This strategy is quite visible especially in the security realm where the US could be seen pressing its partners in the ME not to buy arms from any other country except the US. In addition, the US policy-makers have shown concerns and warned its partners of installing Chinese technology for communication infrastructure. It is reflected from the statement of the then Undersecretary of Defense Kahl warning that, “raising the ceiling too much with Beijing will lower the ceiling with the US” [57].

Analysing the outcome of this last strategy, it has met with limited success as it fell short of appreciating the genuine scope of geopolitical transformations occurring in the region. A good number of Middle Eastern states including KSA, UAE, Qatar etc. are eager to strengthen their relations with the US but are uneasy with the prospect of giving up their ties with other partners. Having more partners than only one helps providing greater degree of liberty and material advantages to the Middle eastern states which the US alone might not offer to them.

### 1.4 A way forward

For the US to sustain its influence in the ME, it must draw a new course. Rather than sticking to the “status quo,” the US should acknowledge the changing geopolitical realities of the region and embrace the evolving multi-aligned arrangements in the ME. By taking leverage of its expansive allies’ network, the US can help facilitating broader ties, embracing new players along with existing relations with China.

Instead of containing or ousting China from the region, it is highly advisable to the US to be a ‘broker.’ This very role of facilitation and arbitration suits to the US natural strengths. This role is compatible and in-line with the previous tactics used by the US in post-war Europe and recently in Asian continent where it has woven its European alliances into Asian engagements. These old strategies can help providing the US to assume “behind-the-scene” role to shape new arrangements which minimise the cost and maximise its reach.

The US needs to develop a robust economic policy primarily which supplement its broader security network in the region. The strong economic footprints of the US in the region will provide its allies and partners with additional opportunities that will result into advancing its clout and engagements in the region. This economic agenda should be framed in such a way that it should emphasize crucial regional priorities where the US usually has an upper hand in comparison with other players such as unconventional energy, climate change, and high-tech [11]. Rather than offering free trade packages, the US should prioritize to build and facilitate new alliance between the ME and the US private sector economic partners [58].

As a starting point, the European partners should be deeply engaged by the US to advance its engagements in the ME in various sectors. Even though Europe is one of the biggest trading partners of the ME, still there is enough room to expand its engagements in the security and diplomatic realms. For example, Baltic Sea littoral states including Finland and Sweden have great exposure in the affairs of maritime security and they usually have same kind of challenges as are faced by the coastal GCC states [59]. This similarity could be utilized as an opportunity for a partnership between the Europe and the ME over awareness of maritime security, piracy attacks, and intelligence sharing for port security. This could also supplement the US efforts in the similar domain in the ME.

Energy is another such area where Europe could be effectively integrated into the ME. Germany, for instance, is interested to advance alternative energy choices with good number of states in the region which will open the avenues for cooperation in renewable energy technologies [60]. This provides the US with an opportunity to work with Germany along with other stakeholders to facilitate joint ventures over green technologies. Canada, being highly equipped with skills in green technology, may be engaged as well. Digital governance is another key area where Europe could be beneficially engaged. The information sharing between Europe and the ME over the matters of digital security and privacy may produce substantial benefits.

The US “brokering strategy” may also be employed to engage its allies in Asia to the ME more strongly. A new cooperation might be encouraged between either Japan and the ME or South Korea and the ME in the industrial and high-tech sectors [61,62]. Cooperation in telecommunication systems between them could also be a great opportunity to enhance regional connectivity and avoid Chinese products in telecommunication [63].

Eventually, all these arrangements would result into accelerating and not impeding the region's drive to multi-alignment by bringing new players and investments. China, nonetheless, would remain actively engaged in the region, while the increasing presence of the US and its allies would help diffusing its clout in ways fruitful to the US. Pundits and critics from around the globe might conceive the growing numbers of stakeholder in the region as a threat to the interests of the US in the ME. But, being a broker, the US would still sustain its central position as a key influencer in the ME.

## 2 Conclusion

The concerns of the US about the increasing clout of Chinese in the region are not completely misplaced given the authoritarian leanings and conflicting interests of China. However, visualizing Chinese growing influence in the ME without taking into consideration the geopolitical shift in the region is counterproductive.

Regional pressures and priorities for multi-alignment will result into pushing regional states to extensively engage with non-US alternatives despite a push by the US. This dooms the very strategy which is aimed at containing or ousting Chinese presence/influence in the ME and the approach based on recreating exclusive US engagements in the region that was the dominant state of affairs during the Cold War. The Middle Eastern states are provided with more agency and leverage due to increasing stakeholders and competition in the region. The future of the ME is ripped with diverse partnerships and the US will be benefitted from quickly embracing that in the long run.

## CRediT authorship contribution statement

**Arfan Mahmood:** Writing – original draft, Visualization, Validation, Supervision, Project administration, Methodology, Investigation, Formal analysis, Data curation, Conceptualization. **Muhammad Usman Askari:** Writing – review & editing, Funding acquisition, Data curation.

## Data availability statement

Data included in article/supp. material is referenced in article.

## Ethics statement

The authors followed ethical guidelines stated in Elsevier's Publishing Ethics Policy.

As this is an original paper, no human participants were involved in this research to provide empirical data.

## Declaration of generative AI and AI-assisted technologies in the writing process

During the preparation of this work, the author(s) did not use AI and AI-assisted technologies in the writing process.

## Funding

This research did not receive any specific grant from funding agencies in the public, commercial, or not-for-profit sectors.

## Declaration of competing interest

The authors declare that they have no known competing financial interests or personal relationships that could have appeared to influence the work reported in this paper.
